# P03 A retrospective review of the safety and efficacy of dalbavancin at Ireland’s largest acute medical hospital

**DOI:** 10.1093/jacamr/dlad066.007

**Published:** 2023-06-14

**Authors:** Peter Conlon, Aisling Murphy, Sarah McKernan, Clare Williamson, Mary Kelly, Ciaran Bannan

**Affiliations:** Department of Infectious Disease, St James’s Hospital, Dublin, Ireland; Department of Infectious Disease, St James’s Hospital, Dublin, Ireland; Department of Infectious Disease, St James’s Hospital, Dublin, Ireland; Department of Pharmacy, St James’s Hospital, Dublin, Ireland; Department of Pharmacy, St James’s Hospital, Dublin, Ireland; Department of Infectious Disease, St James’s Hospital, Dublin, Ireland; School of Medicine Trinity University, Dublin, Ireland

## Abstract

**Background:**

Dalbavancin is a second-generation lipoglycopeptide antibiotic. It is licensed in Ireland and the United Kingdom for the management of acute skin and soft tissue infections. International colleagues report treatment success in its use for patients who are unable to stay in hospital for prolonged periods and not suitable for OPAT.^1^ We reviewed the use of dalbavancin at St. James’s Hospital, Dublin, which is the largest hospital in Ireland with over 700 acute beds. We are located in Dublin’s inner city which experiences significant socio-economic deprivation.

**Methods:**

We used the electronic patient records at St. James’s Hospital to capture all uses of dalbavancin from August 2018 to September 2022. We established baseline demographics, indication, microbiological characteristics, if there was oversight by ID/micro, safety and treatment outcomes. We considered how many hospital days were saved if a patient was changed to dalbavancin and discharged home compared with how long they would have required to stay in hospital to pursue conventional treatment. One hospital day in an acute bed is estimated to cost 800€.

**Results:**

We identified 23 patients who met the inclusion criteria. Baseline demographics are shown in Table 1. The most common indication was endovascular infection (35%) and skin and soft tissue infection (35%) (Figure 1). Most patients were not bacteraemic. *Staphylococcus aureus* was the most common organism identified on blood culture (Figure 2). In total, 322 hospital beds were saved, which equates to 257 600 EUR. No patients had an adverse drug reaction. 100% of cases were discussed with micro or ID.

**Conclusions:**

Dalbavancin is a safe and effective drug. It is another tool that can be used when caring for patients who are experiencing unstable housing and challenging social circumstances. Our experience has found 0 adverse events, 69% of patients experienced treatment success (this increases to 88.8% if we exclude those lost to follow up). This is an important finding considering 17% of indications were considered ‘on label’. Dalbavancin can save money on hospital beds and also reduces non-recyclable plastic associated with repeated doses of IV antibiotics.

**Figure 1.**
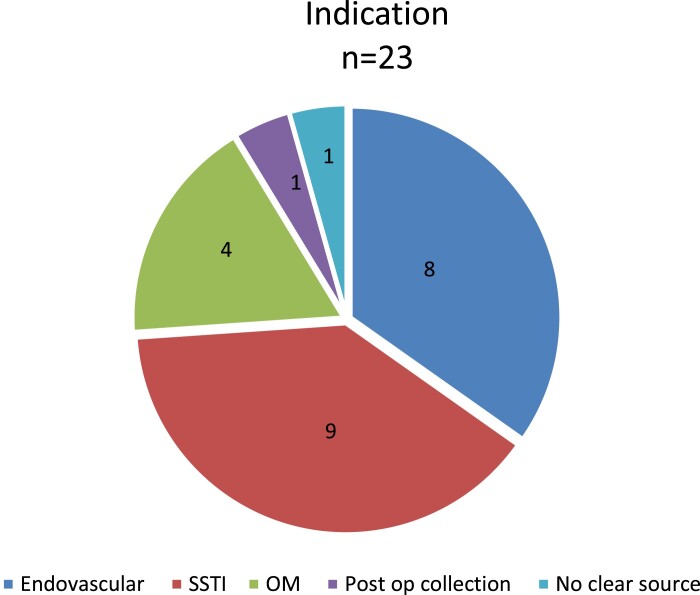
Indication.

**Figure 2.**
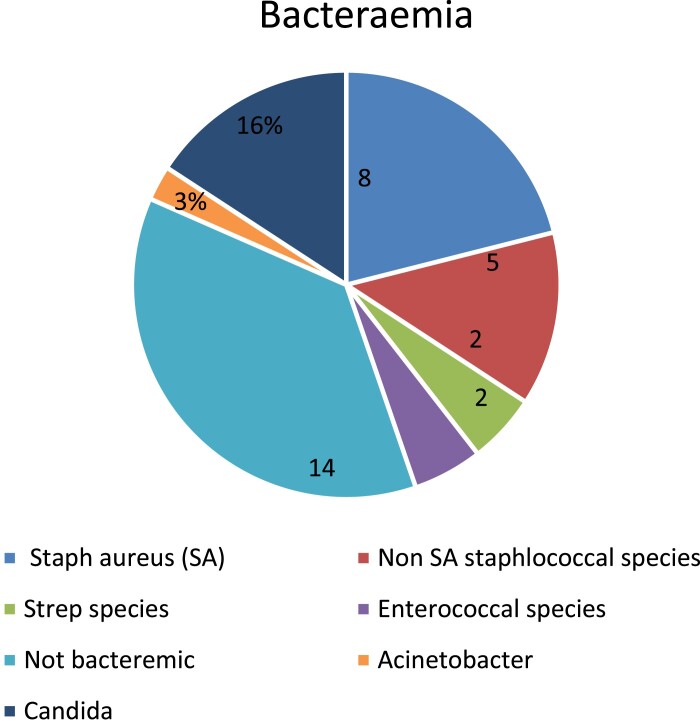
Species.

**Table 1.** Baseline demographics

	*n* (%)
Male	19 (82.6)
Average age, years	42
On label	4 (17.3)
Unstable housing	11 (47.8)
PWID	18 (78.3)
PLWHIV	3 (13)
Followed up	18 (78.3)
Bacteraemic	9 (39)
Adverse drug reaction	0 (0)
Treatment success	16 (69.5)
